# Supplementary material for: Hyperosmotic Stress Response Memory is Modulated by Gene Positioning in Yeast
Source: Cells. 2019 Jun 13;8(6):582. doi: 10.3390/cells8060582 (PMC6627694; doi:10.3390/cells8060582)
Supplement: Supplementary file 1 [file cells-08-00582-s001.zip › supplementary revised/cells-517150 supplementary proofreading.pdf]

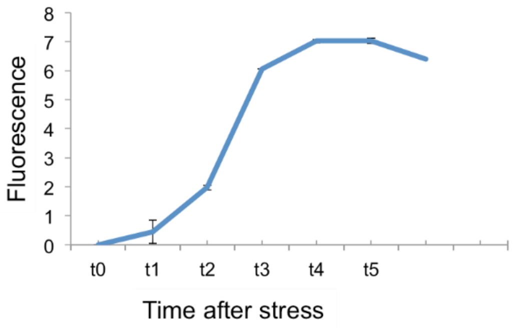

(**a**)

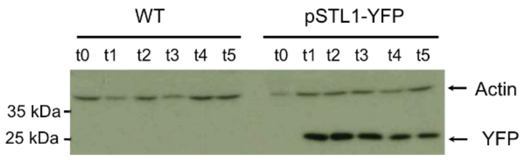

(**b**)

Relative YFP

	t1/t2	t2/t3	t3/t4	t4/t5
WB	0,91	1,12	1,3	1,06
FACS	0,91	1,11	1,2	1,05

(c)

Figure S1. Comparison of relative amounts of pSTL1-YFP by FACS analyses and Western blotting. (a) Cells were sorted by FACS at different time-points after exposure to 1 h stress induced by 1 M sorbitol: 0 min (t0), 50 min (t1), 80 min (t2), 110 min (t3), 140 min (t4), and 170 min (t5). Total fluorescence was normalized to the fluorescence levels before stress. Note the fluorescence response returned to basal levels after 240 min. (b) Total protein extracts were prepared at the same time-points and subjected to Western blotting. Actin was used to normalize the amount of YFP protein expressed. (c). Ratios between YFP protein expression determined by Western blotting and fluorescence intensity measured by FACS.

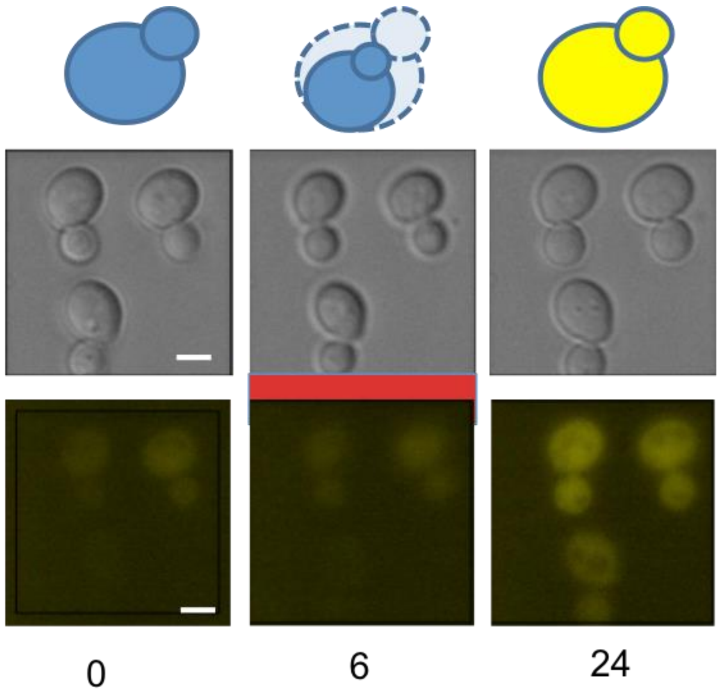

(**a**)

Figure S2. Stress induces cellular compression. (a) Representative images of cells before, during and after stress viewed under Nomarski and fluorescence illumination. A schematic of the cells is shown on the top, the stress is indicated by the red bar. A full video can be found in Video S1

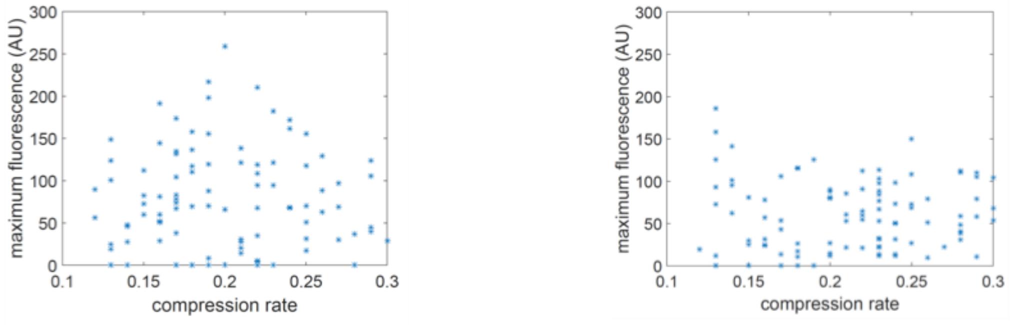

(**a**) (**b**)

Figure S3. Compression ratio of the cells during stress *vs* maximum amplitude of fluorescence. The compression rate was established by segmenting the cells to acquire their area before and during stress and by establishing the ratio between these values: (a) first stress (b) second stress.

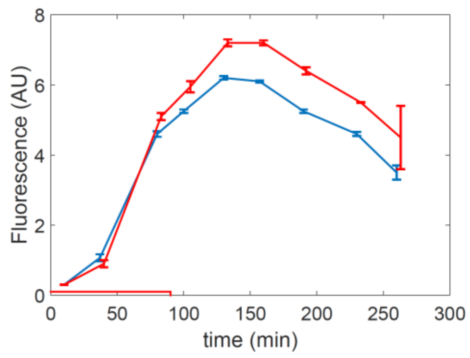

(**a**)

Figure S4. Displaced pSTL1 exhibits reduced activity in response to stress. Fluorescence quantification of the activity of the promoter in response to 90 min hyperosmotic stress for pSTL1 in the endogenous loci (red) and when the promoter was moved to a pericentromeric position (blue). An experiment performed on a distinct clone is presented in Figure 4.

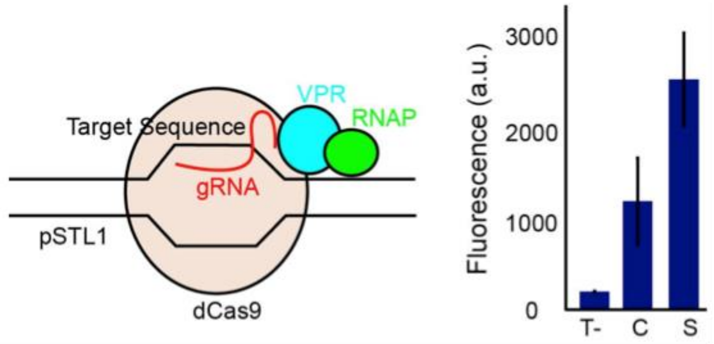

Figure S5. Activation of pSTL1 depends on its genomic position. Sketch of the principle of the dCas9/VPR system. The dCas9 protein is fused to VPR, which enables recruitment of the transcriptional machinery that drives target gene activity. dCAS9/VPR was targeted to the pSTL1 promoter located at its native subtelomere IVR (S) or displaced close to centromere IV using gRNA1 (C) and the corresponding fluorescence intensities were quantified. (T-) cells were not exposed to sorbitol.
